# “Mycophenolate‐Based Immunosuppression After Calcineurin‐Inhibitor Withdrawal in Liver Transplant Recipients is Safe and Results in Long‐Term Improvement in Renal Function”

**DOI:** 10.1111/ctr.70590

**Published:** 2026-06-15

**Authors:** Nicholas Lim, David Vock, Scott Jackson, John Lake

**Affiliations:** ^1^ Division of Gastroenterology, Hepatology and Nutrition University of Minnesota Minneapolis Minnesota USA; ^2^ Division of Gastroenterology and Hepatology University of Rochester Rochester New York USA; ^3^ Transplant Institute University of Rochester Rochester New York USA; ^4^ Division of Biostatistics, School of Public Health University of Minnesota Minneapolis Minnesota USA; ^5^ Analytics Consulting Services, M Health Fairview Minneapolis Minnesota USA; ^6^ Department of Radiation Oncology Stanford University Palo Alto California USA

**Keywords:** calcineurin‐inhibitor, immunosuppression, kidney function, liver transplant

## Abstract

**Introduction:**

Renal dysfunction is an important side effect of calcineurin inhibitors (CNI) and is associated with worse outcomes after liver transplantation (LT). Prior studies on mycophenolate‐based (MMF), CNI‐free immunosuppression regimens have raised concerns about the risk of rejection (ACR). We evaluated the long‐term impact of transition from CNI to MMF‐based immunosuppression on long‐term renal function and other clinical outcomes.

**Methods:**

We performed a single‐center, retrospective review of LT recipients from 1/1/2008 to 12/31/2021 who were transitioned from CNI‐ to MMF‐based immunosuppression for renal dysfunction. Primary outcome was mean change in eGFR at 1‐ and 5‐years after CNI withdrawal: a sequential stratification model was then used to compare changes in eGFR to a control group comprising patients maintained on CNIs at last follow‐up date. Secondary outcomes included first episode of acute cellular rejection (ACR), graft survival and patient survival.

**Results:**

Fifty‐one patients were switched to a MMF‐based immunosuppression regimen at median time of 29.1 months after LT and were then followed for median 93.2 months. Mean age at time of LT was 57.5 years old, 41 (80.4%) patients were male, 44 (86.3%) patients were white and 16 (32.7%) patients had a history of hepatitis C. Model‐based estimates of the increase in eGFR‐CKD‐Epi were 5.7 (95% CI: 2.4, 9.0, *p* = 0.001) mL/min per 1.73 m^2^ and 10.2 (95% CI: 5.0, 15.3, *p* < 0.001) mL/min per 1.73 m^2^ between baseline and 1‐year and between baseline and 5‐years after CNI withdrawal. On multivariable analysis, CNI withdrawal was not associated with an increased risk of ACR (HR 2.22, 95% CI (0.79, 6.23), *p* = 0.13).

**Conclusions:**

The use of MMF‐based immunosuppression after CNI withdrawal results in long‐term improvement in renal function without significantly increasing the risk of ACR. As more LT recipients present with and develop renal dysfunction in the post‐MELD era, conversion to a MMF‐based immunosuppression to preserve kidney function is both safe and effective.

**Abbreviations**
ACR: acute cellular rejection
CKD: chronic kidney disease
CNI: calcineurin‐inhibitor
eGFR: estimated glomerular filtration rate
HCC: hepatocellular carcinoma
HCV: hepatitis C
KT: kidney transplantation
LT: liver transplantation
MASLD: metabolic dysfunction‐associated steatotic liver disease
MELD: model of end‐stage liver disease
MMF: mycophenolate mofetil
MPA: mycophenolic acid
SOT: solid organ transplant
US: United States

## 1 Introduction

Calcineurin inhibitors (CNIs) revolutionized liver transplantation (LT) by improving short‐ and long‐term graft outcomes and are currently the primary immunosuppressive agents used post‐LT [1, 2]. Renal dysfunction is a common side effect of CNIs [3]. Acute CNI‐related nephrotoxicity is related to renal hypoperfusion and afferent arteriolar vasospasm whereas chronic nephrotoxicity is related to glomerulosclerosis, renal tubular atrophy and interstitial fibrosis [4, 5, 6].

Chronic kidney disease (CKD) is more common in LT recipients when compared to other solid organ transplant (SOT) recipients [7]. This has become more prevalent in the years since the model of end‐stage liver disease (MELD) was adopted for use in LT allocation [8]. LT recipients with CKD may go on to develop end‐stage renal disease and ultimately require kidney transplantation (KT) [9]. Furthermore, CKD in LT recipients is associated with an increased risk of mortality [10, 11].

Mycophenolate mofetil (MMF) is a prodrug of mycophenolic acid (MPA), a selective inhibitor of inosine monophosphate dehydrogenase, that was approved for use in LT in the United States (US) in 2000. Enteric‐coated MPA was approved for use in the US in KT recipients in 2004 and is commonly used in LT recipients [12]. MMF and MPA have been used to reduce CNI doses and minimize CNI‐related nephrotoxicity in this patient population [13, 14]. Small, retrospective studies evaluating the transition to MMF/MPA‐based immunosuppression regimens in LT recipients have shown promise with regard to improving kidney function [15, 16, 17]. A recent, large retrospective series of patients gradually transitioned from CNI to MMF monotherapy showed modest improvements in GFR after CNI withdrawal with low rates of rejection [18]. However, concerns about adverse events persist, particularly an increased risk of allograft rejection and failure, and have limited the widespread adoption of MMF/MPA primary immunosuppression regimens [19, 20, 21].

The aims of this study were, therefore, to evaluate long‐term changes in renal function in LT recipients and other clinical outcomes after switching from CNI to a MMF/MPA‐based immunosuppression regimen.

## 2 Methods

### 2.1 Study Population

We performed a retrospective review of LT recipients at our center. Data were collected using the Transplant Information Services database at M Health Fairview. Data unable to be acquired using the database were obtained via manual chart review with approval of the Institutional Review Board at the University of Minnesota (#0704M05324). Data obtained included demographic characteristics, laboratory testing, indication for LT, patient co‐morbidities, donor characteristics, immunosuppression medications and any other concurrent medications including corticosteroids.

We included LT recipients >18 years old who had received a LT from first January 2008 to 31^st^ December 2021. Recipients who had discontinued CNI due to medication non‐adherence, patients who received a simultaneous liver‐kidney transplant, patients on hemodialysis at the time of LT and patients undergoing re‐transplantation were excluded. Patients who died prior to discharge from hospital during the LT admission were also excluded.

Recipients were considered part of the CNI withdrawal group if they were switched from a CNI to MMF/MPA‐based immunosuppression regimen because of renal dysfunction more than three months after their date of LT without a history of acute cellular rejection (ACR). Renal dysfunction was defined as serum creatinine >1.2 mg/dL (the upper limit of normal for our laboratory) for at least two consecutive values, a minimum of three months apart. We did not include patients who switched to a MMF/MPA‐based immunosuppression regimen because of malignancy or neurotoxicity in this group. The control group comprised all other eligible patients.

From March 2009 to March 2017, our center's standard immunosuppression protocol after LT involved an intravenous corticosteroid bolus and interleukin‐2 receptor blocker for induction; a rapid 6‐day corticosteroid taper to no steroids; anti‐metabolite therapy (primarily MMF or MPA) for the first three months, and life‐long calcineurin‐inhibitor therapy. Prior to March 2009, patients did not receive any induction at the time of LT. Since March 2017, basiliximab is no longer part of the standard immunosuppression protocol immediately after LT at our center, but is used at the discretion of the implanting surgeon.

When patients are weaned off CNI at our center, MMF or MPA is started (target dose of MMF 1‐2000 mg or MPA equivalent per day) and CNI dose is halved weekly until discontinuation. Patients are also started on prednisone 5 mg once daily. Liver function tests are checked weekly until CNI has been stopped for three weeks, every two weeks for two checks, monthly for two checks and then back to every two to three months thereafter in accordance with our standard monitoring protocols.

### 2.2 Outcomes

The primary outcome was mean change in estimated glomerular filtration rate (eGFR) at one year and at 5 years after switching from CNI to a MMF/MPA‐based immunosuppression regimen. eGFR was calculated using the CKD Epidemiology collaborative (CKD‐Epi) equation [22]. Changes in eGFR were then compared to a control group comprising patients maintained on CNI. The secondary outcomes were first episode of ACR and overall survival. All cases of suspected ACR were confirmed on liver biopsy.

### 2.3 Statistical Methods

Plots of the mean eGFR and creatinine were created, with the 95% confidence interval also shown. Laboratory values within 3 months of specified time points were used to calculate eGFR.

To compare the difference in the renal function, patient survival, death‐censored graft survival, and survival free from acute rejection episodes (censored at time of death or organ failure) between those withdrawing from CNI and those continuing, we considered a sequential stratification approach. Briefly, each month can be considered as an “experiment” in which we compare the outcomes of those who withdrew from taking CNIs to those who continued. For participants were included in the analysis for that experiment if they were alive with a functioning graft, had not yet experienced the outcome of interest (for time‐to‐event outcomes), and had not previously discontinued CNIs.

For time‐to‐event outcomes, within a given experiment, follow‐up started at the beginning of the landmark and continued until the participant experienced the event of interest or was censored. We pooled the data from all the experiments and fit a proportional hazards model for each outcome with CNI status (withdrawal during the month or continuing) as the key covariate. Because those who discontinue CNIs are prognostically different from recipients continuing CNIs, we adjusted for eGFR at the start of the landmark (or most recent one), age at transplant, sex, race, diabetes, coronary artery disease, HCV, HCC, transplant number in the mortality model and adjusted for age at transplant, sex, race, diabetes, HCV, DCD donor, donor age, and autoimmune disease in the rejection model. In both models, we stratified the analysis by posttransplant month.

To understand the association between CNI withdrawal and subsequent eGFR trajectory, we fit a linear mixed model of the change in eGFR from the start of the experiment pooled across all experiments. Because the change over time may be nonlinear, we included a restricted cubic spline basis expansion of time since start of the experiment. Random effects for recipient and time since start of the experiment were included. This analysis naturally handles the fact that recipients have unequal numbers of follow‐up creatinine measures. From this, we obtained model‐based estimates of the change in eGFR at 1 and 5‐years.

Because participants may continue on CNIs for many months and, thus, contribute to the analysis across multiple experiments, we used robust standard errors. We considered *p*‐value ≤0.05 as statistically significant. All analyses were performed in R, Version 4.4.0 (R Foundation for Statistical Computing, Vienna, Austria).

## 3 Results

### 3.1 Baseline Characteristics

**FIGURE 1:**
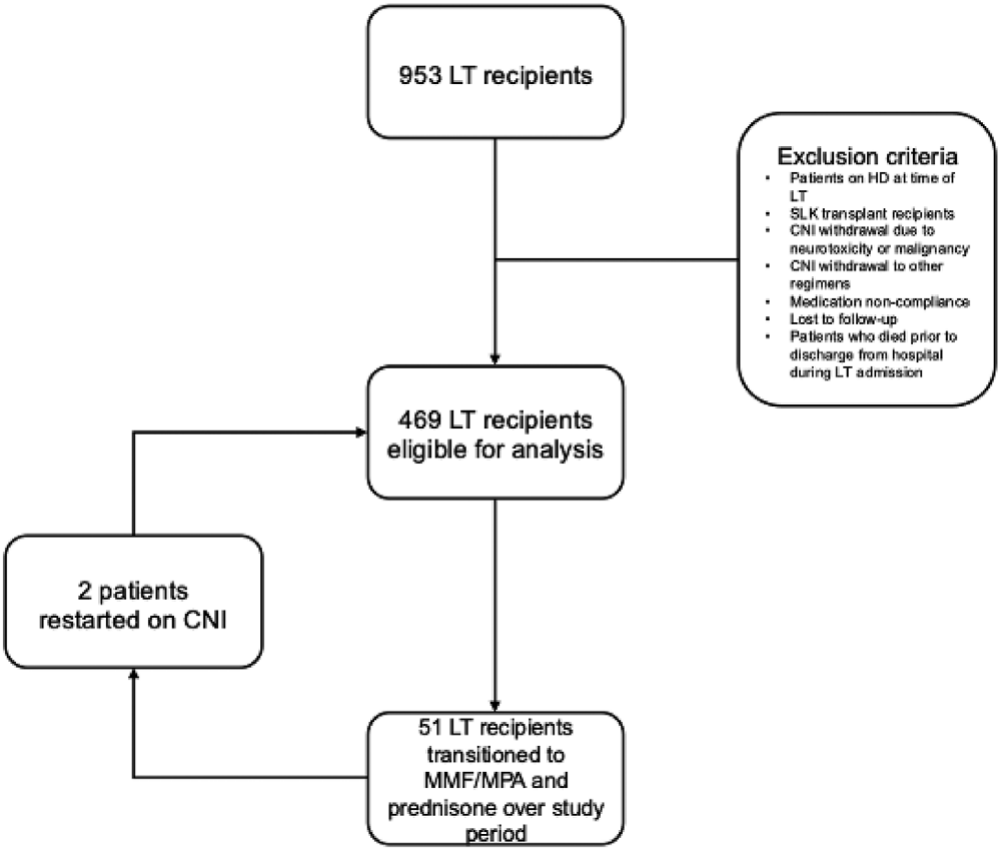
Study cohort flowchart.

Nine hundred and fifty‐three patients underwent LT over the study period, while four hundred and sixty‐nine patients met the inclusion criteria. Fifty‐one patients were switched to a MMF/MPA‐based immunosuppression regimen at median time of 29.1 months after LT and were then followed for median 93.2 months. Of the 51 patients who switched, the mean age at the time of LT was 57.5 years old, 41 (80.4%) patients were male and 44 (86.3%) patients were white. With regard to comorbidities, 11 (21.6%) patients had a history of diabetes and 17 (33.3%) patients had a history of hypertension prior to LT. The median laboratory MELD score at LT was 25.4 Sixteen (32.7%) patients received LT for hepatitis C (HCV), while 22 (43.1%) had a primary or secondary diagnosis of hepatocellular carcinoma (HCC). (Table 1). Two patients resumed CNIs‐ one after transitioning care to the local Veterans Affairs hospital and the other for unknown reasons. (Figure 1).

**TABLE 1:** Baseline characteristics.

	MMF/MPA‐based immunosuppression cohort *n* = 51	CNI at last follow‐up cohort *n* = 469
Median age (at tx), *y*	57.5	53.8
Male gender (%)	41 (80.4)	325 (69.3)
White race (%)	44 (86.3)	376 (80.2)
Median BMI	29.6	28.6
History of coronary artery disease (%)	2 (3.9)	30 (6.4)
History of diabetes mellitus (%)	11 (21.6)	123 (26.2)
History of hypertension (%)	17 (33.3)	111 (23.7)
History of chronic obstructive pulmonary disease COPD (%)	1 (2)	13 (2.8)
Median laboratory MELD score at transplant	25.4	21.4
Median donor age	43	43.3
DCD donor (%)	4 (8.3)	39 (9.6)
CiT, min	368	362.5
WiT, min	52.8	63.8
Etiology of liver disease		
Autoimmune hepatitis	0 (0.0)	13 (2.8%)
Alcohol‐associated liver disease (without HCV)	12 (23.5)	114 (24.3)
HBV	2 (2.0)	19 (4.1)
HCV	12 (23.5)	37 (21.9)
HCV/Alcohol	4 (7.8)	12 (2.6)
HCV/HBV	0 (0.0)	1 (0.2)
MASLD	3 (5.9)	25 (5.3)
Other	18 (35.3)	248 (52.9)
History of HCC (%)	22 (43.1)	179 (38.2)

Abbreviations: BMI, body mass index; CiT, cold ischemic time; DCD, donation after cardiac death; HBV, hepatitis B; HCC, hepatocellular carcinoma; HCV, hepatitis C; MASLD, metabolic dysfunction‐associated steatotic liver disease; MELD, model of end‐stage liver disease; WiT, warm ischemic time.

Four hundred and sixty‐nine patients contributed patient‐time to the control condition. The mean age at LT was 53.8 years, 325 (69.3%) patients were male and 376 (80.2%) patients were white. One hundred and twenty‐three (26.2%) patients had a history of diabetes prior to LT while 111 (23.7%) patients had hypertension prior to LT. The median laboratory MELD score at LT in the control group at was 21.4. Forty‐nine (13.2%) patients had a history of HCV and 149 (40.3%) patients had a history of HCC. (Table 1). Two patients, both in the CNI‐only control group, later underwent KT.

### 3.2 Primary Outcome

**FIGURE 2:**
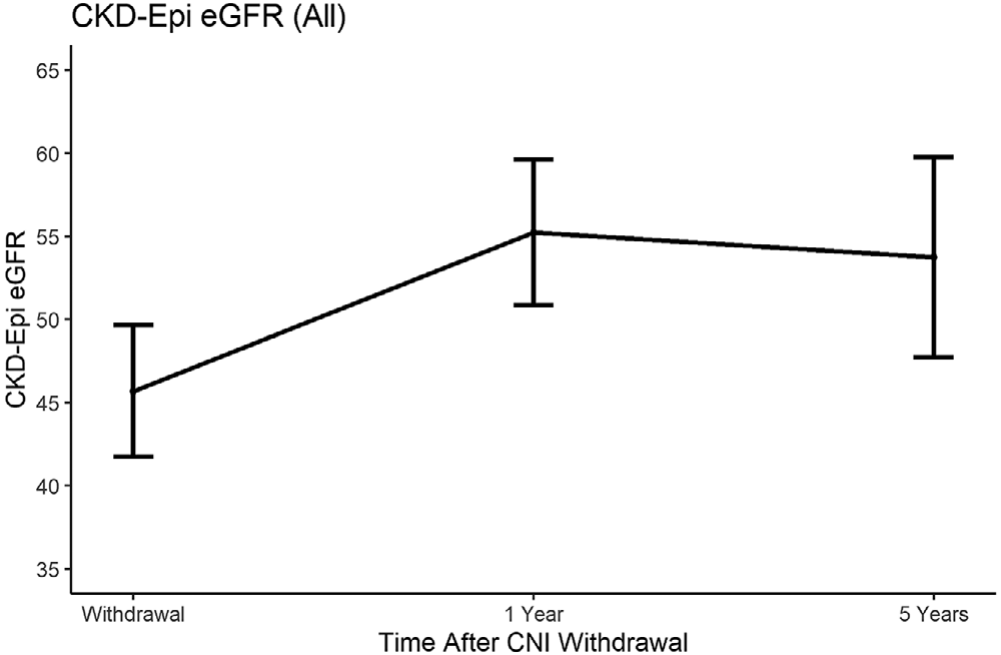
Observed renal function at 0, 1 and 5 years after CNI withdrawal. CNI, Calcineurin‐inhibitor.

In the CNI withdrawal group, the sample mean eGFR‐CKD‐Epi prior to CNI withdrawal was 45.7 (± 12.74) mL/min per 1.73 m^2^ at baseline; 55.2 (± 14.62) mL/min per 1.73 m^2^ at 1‐year after CNI withdrawal and 53.7 (± 13.62) mL/min per 1.73 m^2^ at 5‐years post‐switch among those with available data. (Figure 2). Serum creatinine for the cohort who stopped CNI at baseline was 1.71 (± 0.46) mg/dL, 1.47 (± 0.4) mg/dL at 1‐year after CNI withdrawal and 1.49 (± 0.35) mg/dL at 5‐years post‐switch. (Figure S1).

**FIGURE 3:**
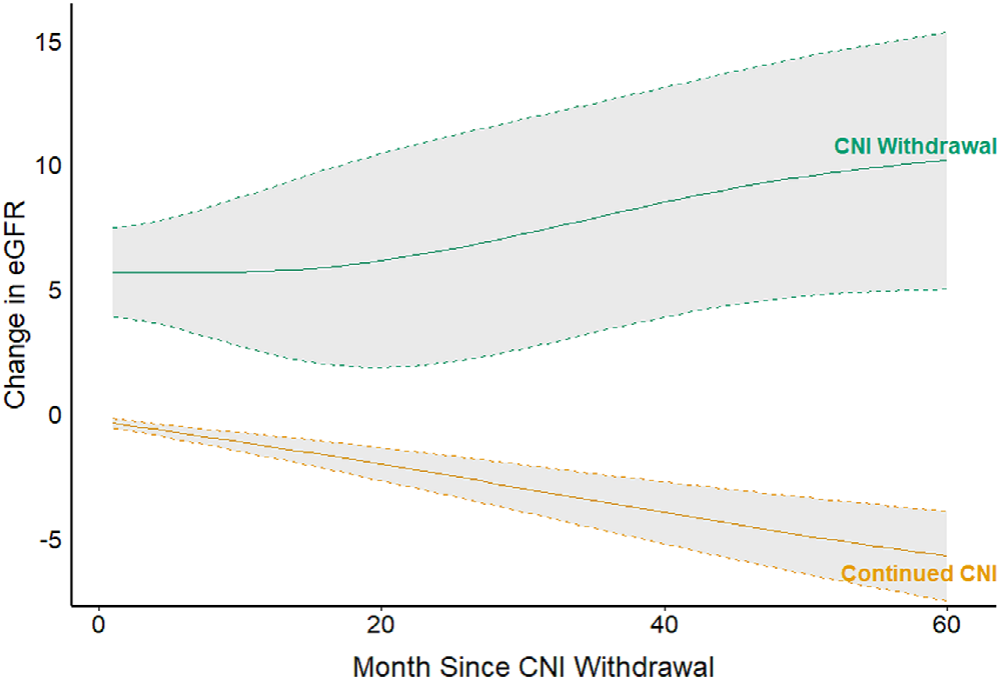
Model‐based estimates of change in eGFR‐CKD‐Epi according to CNI group. CNI, calcineurin‐inhibitor.

Model‐based estimates of the improvement in eGFR‐CKD‐Epi were 5.7 (95% CI: 2.4, 9.0, *p* = 0.001) mL/min per 1.73 m^2^ and 10.2 (95% CI: 5.0, 15.3, *p* < 0.001) mL/min per 1.73 m^2^ between baseline and 1‐year and between baseline and 5‐years after CNI withdrawal. This compares to a decrease of 1.3 (95% CI: 0.9, 1.7, *p* < 0.001) mL/min per 1.73 m^2^ and 5.6 (95% CI: 3.8, 7.4, *p* < 0.001) mL/min per 1.73 m^2^ between baseline and 1‐year and between baseline and 5‐years among those continuing CNIs. (Figure 3).

### 3.3 Secondary Outcomes

#### 3.3.1 ACR

**FIGURE 4:**
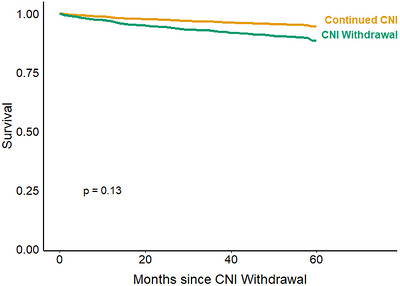
Adjusted ACR‐free survival according to CNI group. CNI, calcineurin‐inhibitor.

In total, ten patients developed ACR in the follow‐up period after CNI withdrawal in the study cohort, while 93 patients developed ACR in the cohort of patients who remained on CNI until last follow‐up date. On multivariate analysis, there was no significant association with CNI withdrawal to a MMF/MPA‐based immunosuppression regimen and development of ACR (HR 2.22, 95% CI (0.79, 6.23), *p* = 0.13). Higher eGFR and older age at transplant were associated with lower risk of ACR. (Table 2). Only ten patients developed graft failure without subsequent death between the two cohorts over the entire study period. Estimated adjusted ACR‐free survival comparing CNI withdrawal to continuing CNI is given in Figure 4.

**TABLE 2:** Multivariable analysis for first episode of ACR.

Clinical variable	HR (95% CI)	*p*‐value
CNI withdrawal	2.22 (0.79, 6.23)	0.13
Mean eGFR	0.97 (0.96, 0.99)	0.003
Age at transplant	0.93 (0.89, 0.96)	<0.01
Male gender	1.94 (0.56, 6.77)	0.3
White race	0.51 (0.19, 1.37)	0.18
History of hepatitis C	1.16 (0.43, 3.11)	0.77
Donation after circulatory death	2.34 (0.33, 16.59)	0.4
Donor age	0.97 (0.94, 1.01)	0.1
History of autoimmune hepatitis	1.62 (0.12, 21.9)	0.72

#### 3.3.2 Overall Survival

**FIGURE 5:**
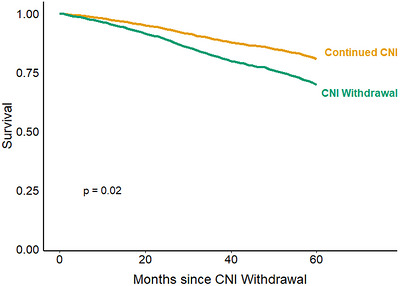
Adjusted overall survival according to CNI group.

There were 25 deaths after CNI withdrawal over the study period while 50 patients died in the cohort of patients who remained on CNI. In the CNI withdrawal group, the causes of death were cardiovascular disease (*n* = 2, 8%), infection (*n* = 3, 12%), cancer‐related (*n* = 4, 16%), liver‐related (*n* = 3, 12%), respiratory disease (*n* = 4, 16%), other causes (*n* = 5, 20%) and unknown in four (16%) patients. In the control group, causes of death were cardiovascular (*n* = 6, 12%), infection (*n* = 8, 16%), cancer‐related (*n* = 26, 52%), liver‐related (*n* = 4, 8%), other causes (*n* = 1, 2%) ad unknown causes (*n* = 2, 4%). On multivariate analysis, CNI withdrawal was associated with higher risk of death (HR 1.72, 95% CI (1.09, 2.73), *p* = 0.02). Higher eGFR was associated with a lower risk of death. (Table 3). Estimated adjusted patient survival comparing CNI withdrawal to continuing CNI is given in Figure 5.

**TABLE 3:** Multivariable analysis for overall survival.

Clinical variable	HR (95% CI)	*p*‐value
CNI withdrawal	1.72 (1.09, 2.73)	0.02
Mean eGFR	0.98 (0.97, 0.99)	<0.01
Age at transplant	1.01 (0.98, 1.04)	0.62
Male gender	1.03 (0.53, 2.02)	0.93
White race	0.7 (0.34, 1.45)	0.34
History of diabetes mellitus	1.09 (0.54, 2.19)	0.82
History of coronary artery disease	1.16 (0.42, 3.23)	0.77
History of hepatitis C	1.1 (0.6, 2.02)	0.76
History of HCC	1.54 (0.82, 2.92)	0.18

## 4 Discussion

Among nonrenal SOT recipients, LT recipients have the highest rates of CKD, which is associated with poorer overall survival [7]. This has been attributed, in part, to higher rates of LT recipients with acute kidney injury at the time of LT since incorporation of MELD into liver allocation [8]. In addition, rates of CKD are expected to rise further both as existing LT recipients grow older and as more patients with diabetes undergo LT for metabolic dysfunction‐associated steatotic liver disease (MASLD), making the results from our study increasingly important today [23, 24].

Our study shows that transitioning to an MMF/MPA‐based immunosuppression regimen from CNI in LT recipients leads to a sustained improvement in renal function, most likely related to reduction of vasoconstriction of the afferent and efferent arterioles in the kidney [6]. Older studies have reported short‐term improvements in renal function after CNI withdrawal [25, 26, 27]. One recent study from Spain did demonstrate a statistically (but perhaps not clinically) significant improvement in median GFR over a median follow‐up time of 68 months. Patients in this cohort were switched to MMF/MPA‐based immunosuppression regimens for several indications over a longer median transition period of 18 months, which may have negatively impacted the potential for improvement of kidney function. Notably, this cohort did not contain any patients with MASLD so the study generalizability may also be limited given the changing epidemiology of LT in the West [18].

In our study, eGFR improved soon after CNI withdrawal, consistent with prior CNI withdrawal or CNI minimization studies [28, 29, 30], and persisted 5 years later. However, a mild, nonsignificant decline in eGFR was seen overall between 1‐ and 5‐years after CNI withdrawal, consistent with changes seen with aging and HCV as the primary liver disease of a notable proportion of our cohort [31, 32]. When looking at the model‐based estimate, patients in the CNI withdrawal group experienced a gradual, sustained improvement in eGFR over the follow‐up time relative to their baseline eGFR. In comparison, patients maintained on CNIs experienced a persistent steady decline in renal function. Although the improvements in renal function observed in our study appear modest, they may be beneficial to LT recipients as even small differences in eGFR have been associated with an increased risk of cardiovascular mortality in the general population [33].

In our cohort, CNI withdrawal to MMF/MPA‐based immunosuppression was not associated with a significantly increased risk of ACR. Earlier studies describing transition to MMF/MPA‐based immunosuppression in LT recipients reported high rates of ACR with some patients even requiring repeat LT [19, 20, 21]. More recent studies have not reported these problems, although patients were transitioned to MMF/MPA‐based immunosuppression several years after LT [18, 34, 35]. At our center, patients with a prior history of ACR cannot start CNI withdrawal, which may in part explain the unchanged risk of ACR despite transitioning to MMF/MPA‐based immunosuppression earlier than most reports. In our cohort, increased age at LT was associated with lower risk of ACR in our cohort, consistent with prior studies of rejection in older patients [36, 37]. As the risk of CKD after LT increases with age, older patients in particular may stand to gain the most benefit from transitioning to MMF/MPA‐based immunosuppression with less risk to their allograft function [38].

CNI withdrawal was associated with increased mortality on multivariate analysis in our study. CNIs are the current gold standard for immunosuppression in LT recipients due to their positive effect on both patient and graft survival [1, 2]. Therefore, the negative impact on mortality observed from CNI withdrawal in our study was unsurprising, particularly as CNIs were discontinued due to renal dysfunction, which is associated with higher mortality in LT recipients [10, 11]. Conversely, increases in eGFR were associated with lower mortality, supporting this theory. Finally, while it is possible that an MMF/MPA‐based immunosuppression regimen itself may directly increase mortality, previous studies have not shown this association [34, 35]. A large study using data from the Scientific Registry of Transplant Recipients reported that the use of MMF is associated with lower mortality in LT recipients both with and without hepatitis C [39]. To date, the only association between MMF and higher mortality has been observed in LT recipients with COVID‐19 infection [40].

A strength of our analysis is the use of a sequential stratification or landmarking approach over a proportional hazards model or longitudinal model with time‐varying covariates. Briefly, time‐varying covariates may “adjust away” important associations between CNI withdrawal and clinical outcomes by updating covariates in the model which are intermediate between CNI withdrawal and outcomes. For example, including eGFR as a time‐varying covariate in the model for patient survival may “block” or “adjust away” some of the effect of CNI withdrawal as the primary beneficial effect of CNI withdrawal is to increase renal function. However, excluding eGFR from the model may lead to confounding by indication because reduced eGFR is the primary indication for CNI withdrawal.

The limitations of our study include the single‐center, retrospective design which may limit the applicability of our findings. We cannot comment on severity of rejection as we do not have consistent data on severity of episodes of ACR (using Banff criteria) on liver biopsy specimens obtained prior to 2018. We have not included extensive data on side effects, as these have been well described previously and prior studies of MMF/MPA‐based immunosuppression have not reported any significant differences regarding gastrointestinal symptoms or pancytopenia [34, 35, 41]. We also acknowledge that both cardiovascular disease and de novo malignancy are important causes of long‐term morbidity and mortality but opted to focus on the long‐term challenges with kidney function in LT recipients. Next, we did not have enough cases to assess the impact of timing of CNI withdrawal on renal function. In patients transitioned to mammalian target of rapamycin inhibitors from CNI at more than 12 months after LT, improvement in kidney function is less common [9]. However, our cohort was transitioned at a median of 29.1 months after LT, suggesting that timing of CNI withdrawal may be less important in patients transitioned to a MMF/MPA‐based immunosuppression regimen. Polytransfusion was not considered in our analysis as it has not been consistently shown to be a risk factor for kidney dysfunction in LT recipients [42, 43]. As with any study evaluating immunosuppression, patients may not always have been at target trough levels despite strict adherence to center protocols, particularly when levels were being checked less frequently. Finally, due to our study design, we were unable to adjust for an era effect when patients transplanted more recently were taking lower doses of immunosuppression which may have affected kidney function.

In summary, CNI withdrawal and transition to MMF/MPA‐based immunosuppression in LT recipients results in long‐term improvement in kidney function without significantly increasing the risk of ACR. As the incidence of CKD in LT recipients increases, CNI withdrawal could reduce the progression to end‐stage kidney disease and the future need for KT. Our findings should reassure providers who are apprehensive about transitioning their patients to MMF/MPA‐based immunosuppression regimens due to concerns about ACR. Future studies should evaluate the impact of timing of CNI withdrawal when transitioning to MMF or MPA, the impact on cardiometabolic comorbidities and de novo malignancy, and the potential for CNI withdrawal in LT recipients with a prior history of rejection.

## Author Contributions

**Nicholas Lim**: study concept, data collection, data review, manuscript drafting, manuscript review, manuscript revision. **David Vock**: statistical methodology, data collection, data analysis. **Scott Jackson**: data collection, data analysis. **John Lake** study concept, data review, critical manuscript review, critical manuscript revision.

## Conflicts of Interest

The authors declare no conflicts of interest.

## Supporting information




**Supplemental Figure 1**: Serum Creatinine at 0, 1 and 5 years after CNI withdrawal.

## Data Availability

The data that support the findings of this study are available on request from the corresponding author. The data are not publicly available due to privacy or ethical restrictions.
