# Supplementary material for: “Mycophenolate‐Based Immunosuppression After Calcineurin‐Inhibitor Withdrawal in Liver Transplant Recipients is Safe and Results in Long‐Term Improvement in Renal Function”
Source: Clin Transplant. 2026 Jun 15;40(6):e70590. doi: 10.1111/ctr.70590 (PMC13267676; doi:10.1111/ctr.70590)
Supplement: Supplementary file 1 — Supplemental Figure 1: Serum Creatinine at 0, 1 and 5 years after CNI withdrawal. [file CTR-40-e70590-s001.docx]

**Supplemental Figures**

Supplemental Figure 1- Serum Creatinine at 0, 1 and 5 years after CNI withdrawal


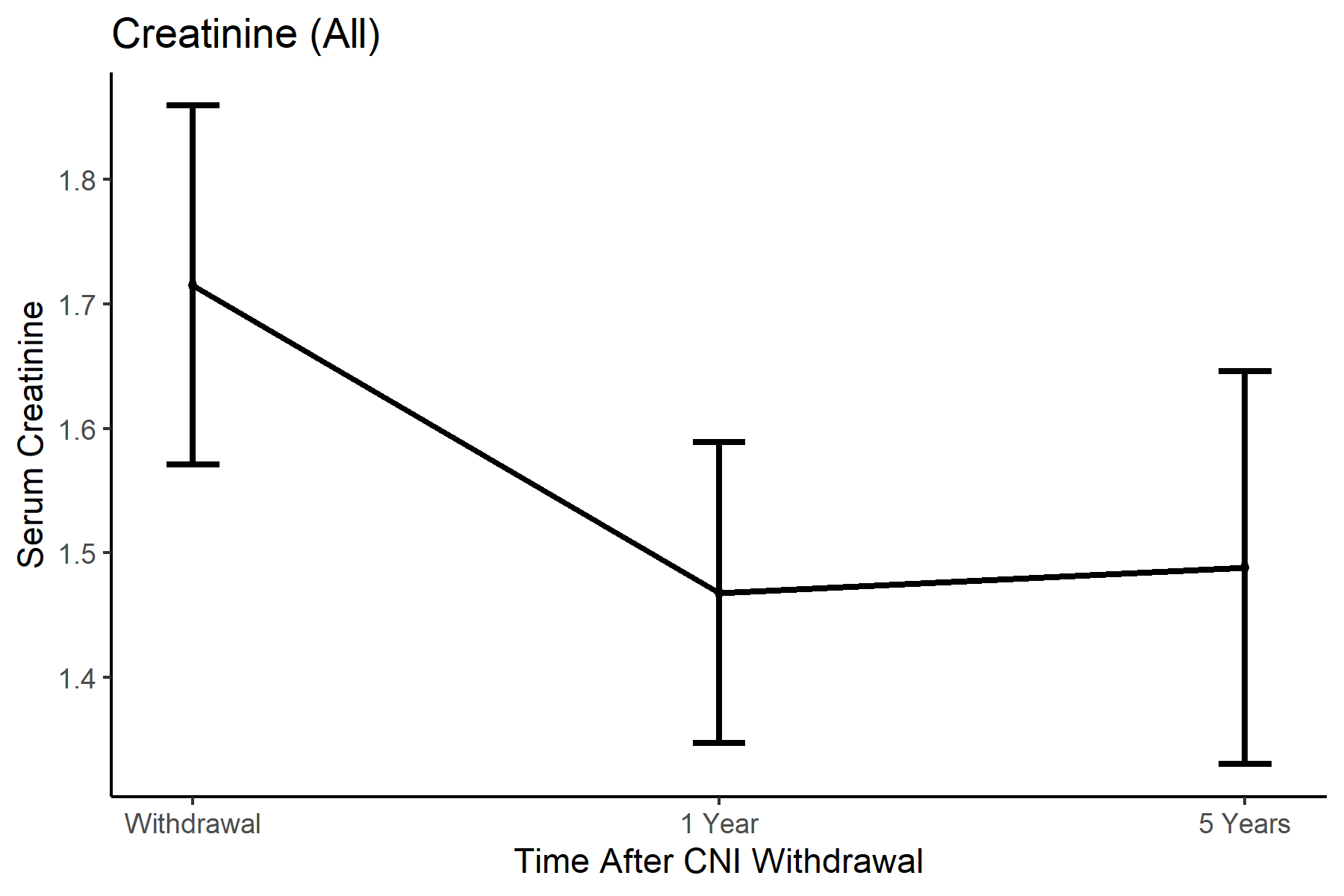


CNI= Calcineurin-inhibitor
